# GPR183 Regulates Interferons, Autophagy, and Bacterial Growth During *Mycobacterium tuberculosis* Infection and Is Associated With TB Disease Severity

**DOI:** 10.3389/fimmu.2020.601534

**Published:** 2020-11-06

**Authors:** Stacey Bartlett, Adrian Tandhyka Gemiarto, Minh Dao Ngo, Haressh Sajiir, Semira Hailu, Roma Sinha, Cheng Xiang Foo, Léanie Kleynhans, Happy Tshivhula, Tariq Webber, Helle Bielefeldt-Ohmann, Nicholas P. West, Andriette M. Hiemstra, Candice E. MacDonald, Liv von Voss Christensen, Larry S. Schlesinger, Gerhard Walzl, Mette Marie Rosenkilde, Thomas Mandrup-Poulsen, Katharina Ronacher

**Affiliations:** ^1^ Translational Research Institute–Mater Research Institute, The University of Queensland, Brisbane, QLD, Australia; ^2^ DSI-NRF Centre of Excellence for Biomedical Tuberculosis Research, South African Medical Research Council Centre for Tuberculosis Research, Division of Molecular Biology and Human Genetics, Faculty of Medicine and Health Sciences, Stellenbosch University, Cape Town, South Africa; ^3^ School of Chemistry and Molecular Biosciences, The University of Queensland, St Lucia, QLD, Australia; ^4^ Australian Infectious Diseases Research Centre, The University of Queensland, Brisbane, QLD, Australia; ^5^ Department of Biomedical Sciences, University of Copenhagen, Copenhagen, Denmark; ^6^ Host-Pathogens Interactions Program, Texas Biomedical Research Institute, San Antonio, TX, United States

**Keywords:** Mycobacterium tuberculosis, diabetes, oxysterols, 7α,25-dihydroxycholesterol, GPR183, EBI2, host-direct therapies, autophagy

## Abstract

Oxidized cholesterols have emerged as important signaling molecules of immune function, but little is known about the role of these oxysterols during mycobacterial infections. We found that expression of the oxysterol-receptor GPR183 was reduced in blood from patients with tuberculosis (TB) and type 2 diabetes (T2D) compared to TB patients without T2D and was associated with TB disease severity on chest x-ray. GPR183 activation by 7*α*,25-dihydroxycholesterol (7*α*,25-OHC) reduced growth of *Mycobacterium tuberculosis* (Mtb) and *Mycobacterium bovis* BCG in primary human monocytes, an effect abrogated by the GPR183 antagonist GSK682753. Growth inhibition was associated with reduced IFN-β and IL-10 expression and enhanced autophagy. Mice lacking GPR183 had significantly increased lung Mtb burden and dysregulated IFNs during early infection. Together, our data demonstrate that GPR183 is an important regulator of intracellular mycobacterial growth and interferons during mycobacterial infection.

## Introduction

Patients with tuberculosis and type 2 diabetes (TB–T2D) co-morbidity have increased bacterial burden and more severe disease, characterized by higher sputum smear grading scores and greater lung involvement on chest x-ray compared to TB patients without T2D (1, 2). TB–T2D patients are also more likely to fail TB therapy and to relapse (3). The reason for the increased disease severity has largely been attributed to hyperglycemia-mediated immune dysfunction, but hyperglycemia alone does not fully explain these observations (3, 4). We recently showed that independent of hyperglycemia, cholesterol concentrations in T2D patients vary greatly across different ethnicities (5). However, how cholesterol and its metabolites contribute to *Mycobacterium tuberculosis* (Mtb) infection outcomes remains to be elucidated.

To gain novel insights into the underlying immunological mechanisms of increased susceptibility of T2D patients to TB and to identify novel targets for host-directed therapy (HDT), we performed whole blood transcriptomic screens on TB patients with and without T2D and identified differential regulation of the transcript for oxidized cholesterol-sensing G protein-coupled receptor (GPCR), GPR183. Also known as Epstein Barr virus-induced gene 2 (EBI2), GPR183 is primarily expressed on cells of the innate and adaptive immune system (6–8). Several oxysterols can bind to GPR183 with 7*α*,25-hydroxycholesterol (7*α*,25-OHC) being the most potent endogenous agonist (6, 9, 10). GPR183 has been studied mainly in the context of viral infections (11), immune cells (6, 7, 9, 12–18), and astrocytes (19, 20); it facilitates the chemotactic distribution of lymphocytes, dendritic cells and macrophages to secondary lymphoid organs (12, 15, 16, 21, 22). Little is known about the biological role of GPR183 in the context of bacterial infections, including TB. We show here that GPR183 is a key regulator of intracellular bacterial growth and type-I IFN production during mycobacterial infection and reduced GPR183 expression is associated with increased TB disease severity.

## Methods

### Study Participants

TB patients and their close contacts were recruited at TB clinics outside Cape Town (South Africa). TB diagnosis was made based on positive GeneXpert MTB/RIF (Cepheid; California, USA) and/or positive MGIT culture (BD BACTED MGIT 960 system, BD, New Jersey, USA) and abnormal chest x-ray. Chest x-rays were scored, based on Ralphs score (23), by two clinicians independently. Participants with LTBI were close contacts of TB patients who tested positive on QuantiFERON-TB Gold in tube assay (Qiagen, Hilden, Germany). All study participants were screened for T2D based on HbA1c ≥6.5% and random plasma glucose ≥200 mg/dl or a previous history of T2D. Further details are available in the **Supplementary Materials**.

### RNA Extractions and Nanostring Analysis

Total RNA was extracted from cell pellets collected in QuantiFERON-TB gold assay tubes without antigen using the Ribopure Ambion RNA isolation kit (Life Technologies, California, USA) and eluted RNA treated with DNase for 30 min. Samples with a concentration of ≥20 ng/µl and a 260/280 and 260/230 ratio of ≥1.7 were analyzed at NanoString Technologies in Seattle, Washington, USA. Differential expression of 594 genes, including 15 housekeeping genes, was performed using the nCounter GX Human Immunology kit V2. NanoString RCC data files were imported into the nSolver 3 software (nSolver Analysis software, v3.0), and gene expression was normalized to housekeeping genes.

### Cell Culture

Peripheral blood mononuclear cells (PBMCs) were obtained from healthy donor blood by Ficoll-Paque (GE Healthcare, Illinois, USA) gradient centrifugation and monocytes (MNs) isolated using the Pan Monocyte Isolation kit (Miltenyi Biotec, Bergisch Gladbach, Germany), with >95% purity assessed by flow cytometry. MNs were plated onto Poly-D-lysine coated tissue culture plates (1.3 × 10^5^ cells/well) and rested overnight at 37°C/5%CO_2_ in RPMI-1640 medium supplemented with 10% heat-inactivated human AB serum (Sigma Aldrich, Missouri, USA), 2 mM L-glutamine and 1 mM sodium pyruvate before infection. THP-1 cells (ATCC #TIB-202) were differentiated with 25 ng/ml PMA for 48 h and rested for 24 h prior to infection.

### *In Vitro* Mtb (H_37_R_v_)/*M. bovis* (BCG) Infection

Mtb H_37_R_v_ or *M. bovis* BCG single cell suspensions were added at a multiplicity of infection (MOI) of 1 or 10 with/without 100 nM 7*α*,25-dihydroxycholesterol (Sigma Aldrich) and with/without 10 µM GSK682753 (Focus Bioscience, Queensland, Australia), followed by 2 h incubation at 37°C/5%CO_2_ to allow for phagocytosis. Non-phagocytosed bacilli were removed by washing each well twice in warm RPMI-1640 containing 25 mM HEPES (Thermo Fisher Scientific). Infected cells were incubated (37°C/5%CO_2_) in medium with/without GPR183 agonist and/or antagonist and CFUs determined after 48 h.

To quantify bacterial growth over time, CFUs at 48 h were normalized to uptake at 2 h. Percentages of mycobacterial growth were determined relative to untreated cells. For RNA extraction, MNs were lysed by adding 500 µl of TRIzol reagent. Further details are provided in the supplementary information.

### Western Blotting

THP-1 cells were infected with BCG with/without 100 nM 7*α*,25-OHC and with/without 10 µM GSK682753 and lysed at 6 or 24 h post infection (p.i.) in ice-cold RIPA buffer (150 mM sodium chloride, 1.0% Triton X-100, 0.5% sodium deoxycholate, 0.1% SDS, 50 mM Tris, pH 8.0; Thermo Fisher Scientific), supplemented with complete Protease Inhibitor Cocktail (Sigma Aldrich) (120 µl RIPA/1 × 10^6^ Cells). Protein concentrations were determined using Pierce BCA Protein Assay Kit (Thermo Fisher Scientific) as per manufacturer’s protocol. 10 µg of protein per sample was loaded on NovexTM 10–20% Tris-Glycine protein gels (Thermo Fisher Scientific) and transferred onto iBlot2 Transfer Stacks PVDF membrane (Thermo Fisher Scientific). Membranes were blocked with Odyssey Blocking buffer (Milennium Science, Victoria, Australia) for 2 h, probed with rabbit anti-human LC3B (1:1,000, Sigma L7543) and rabbit anti-human GAPDH (1:2,500, Abcam 9485) overnight, followed by detection with goat anti-rabbit IgG DyLight 800 (1:20,000; Thermo Fisher Scientific). Bands were visualized using the Odyssey CLx system (LI-COR Biosciences, Nebraska, USA) and analyzed with Image Studio Lite V5.2 (LI-COR Biosciences).

### Immunofluorescence

Differentiated THP-1 cells were seeded onto a poly-D-lysine coated, 96-well glass-bottom black tissue culture plate (4.5 × 10^4^ cells/well) and kept in RPMI-1640 medium minus phenol red (Thermo Fisher Scientific) supplemented with 10% heat-inactivated FBS at 37°C/5% CO_2_. Cells were infected with BCG at a MOI of 10, with/without 100 nM 7*α*,25-OHC, with/without 10 µM GSK682753 for 2 h, washed and incubated for a further 4 h with agonists and antagonists. Cells were then fixed with 4% paraformaldehyde in PBS for 15 min, permeabilized with 0.05% saponin (Sigma Aldrich) for 20 min and blocked with 1% BSA, 0.05% saponin (Sigma Aldrich) for 1 h. Cells were immunolabeled with rabbit anti-human LC3B (ThermoFisher L10382; 1:1,000), 0.05% saponin at room temperature for 1 h followed by Alexa FluorTM 647 goat anti-rabbit IgG (ThermoFisher A21245; 1:1,000), 0.05% saponin at room temperature for 1 h followed by nuclear staining with Hoechst 33342 (Thermo Fisher Scientific 62249; 1:2,000) for 15 min. Cells were washed, and confocal microscopy was performed using the Olympus FV3000, 60× magnification. Images obtained were analyzed with the ImageJ software (24).

### Murine GPR183 KO *vs* WT Model

Equal numbers of male and female C57BL/6 WT and Gpr183^tm1Lex^ (age 18–20 weeks, 10 mice per group/timepoint) were aerosol infected with 300 CFU Mtb H_37_R_v_ using an inhalation exposure system (Glascol). At 2- and 5-weeks post infection, lungs and blood were collected for RNA and CFU determination. Formalin-fixed lung lobes were sectioned and examined microscopically and scored by a veterinary pathologist. Further details are available in the supplementary information.

### Statistical Analysis

Statistical analysis was performed using GraphPad Prism v.7.0.3 (GraphPad Software). *T*-test and Wilcoxon’s test were used to analyze Nanostring data. Mann–Whitney *U* test and *t*-test were used to analyze *in vitro* infection, qPCR, and ELISA data. Data are presented as means ± SEM. Statistically significant differences between two groups are indicated in the figures as follows ns, *P* > 0.05; *, P < 0.05; **, P < 0.01; ***, P < 0.001; ****, P < 0.0001.

### Ethics Statement

The human studies were approved by the Institutional Review Board of Stellenbosch University (N13/05/064 and N13/05/064A) and all study participants signed pre-approved informed consent documents prior to enrolment into the studies. All animal studies were approved by the Animal Ethics Committee of the University of Queensland (MRI-UQ/596/18) and conducted in accordance with the *Australian Code for the Care and Use of Animals for Scientific Purposes*.

## Results

### Blood GPR183 mRNA Expression Is Reduced in Patients With TB–T2D Compared to TB Patients Without T2D

Blood was obtained from the study participants with latent TB infection (LTBI, n = 11), latent TB infection with T2D (LTBI + T2D, n = 14), active pulmonary TB disease (TB, n = 9), and active pulmonary TB disease with T2D (TB + T2D, n = 7). Total RNA was extracted and NanoString analyses performed. Among genes differentially expressed between TB and TB + T2D we identified a single GPCR, GPR183. We focused on GPR183 as GPCRs are *bona fide* drug targets due to their importance in human pathophysiology and their pharmacological tractability.

**Figure 1:**
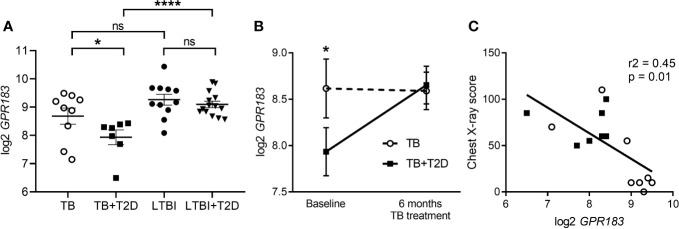
GPR183 mRNA expression in patients with active and latent TB infection with or without T2D. Total RNA was isolated from whole blood incubated overnight in QuantiFERON-TB Gold. GPR183 mRNA expression was determined and normalized to reference genes using the NanoString technology. GPR183 expression in whole blood of **(A)** TB (n = 9) and TB + T2D (n = 7) patients, LTBI (n = 11) and LTBI + T2D (n = 14) patients, Wilcoxon test. **(B)** TB (n = 9) and TB + T2D (n = 7) patients at baseline and 6 month’s treatment, t-test. **(C)** Correlation between GPR183 expression and chest x-ray score, TB + T2D patients (n = 7) filled squares, TB patients (n =8) open circles. Data are presented as means ± SEM; not significance (ns) P > 0.05; *P ≤ 0.05; ****P ≤ 0.0001.

GPR183 expression was significantly down-regulated at diagnosis (p = 0.03, *t*-test) in blood from TB + T2D patients compared to TB patients without T2D (**Figure 1A**). The reduced GPR183 expression was not driven by diabetes *per se*, as there were no differences in GPR183 expression between LTBI and LTBI + T2D (**Figure 1A**). After 6 months, at the end of successful TB treatment, we saw GPR183 expression significantly increased (p = 0.0156) in TB + T2D patients to a level comparable to the TB patients without T2D (**Figure 1B**). Therefore, we speculated that blood GPR183 expression is associated with extent of TB disease, which is frequently more severe in T2D patients. We indeed determined an inverse correlation between GPR183 mRNA expression in the blood and TB disease severity on chest x-ray (**Figure 1C**).

In order to identify which cell type is associated with decreased expression of GPR183 in the blood, we performed flow cytometry analysis for GPR183 expression on PBMCs from TB patients with and without T2D. We investigated GPR183 expression on CD4+ and CD8+ T-cells, B cells, dendritic cells, NK cells and monocytes. We found that the only cell type with a significant reduction in GPR183 positivity in TB + T2D *vs.* TB, both in terms of frequency and median fluorescent intensity, was the non-classical monocyte population (**Supplementary Figures 1A, B**). The frequencies of GPR183 + non-classical monocytes from LTBI and LTBI + T2D were not significantly different (**Supplementary Figure 1C**). We therefore next investigated whether GPR183 plays a role in the innate immune response during Mtb infection.

### Oxysterol-Induced Activation of GPR183 Reduces Intracellular Mycobacterial Growth

**Figure 2:**
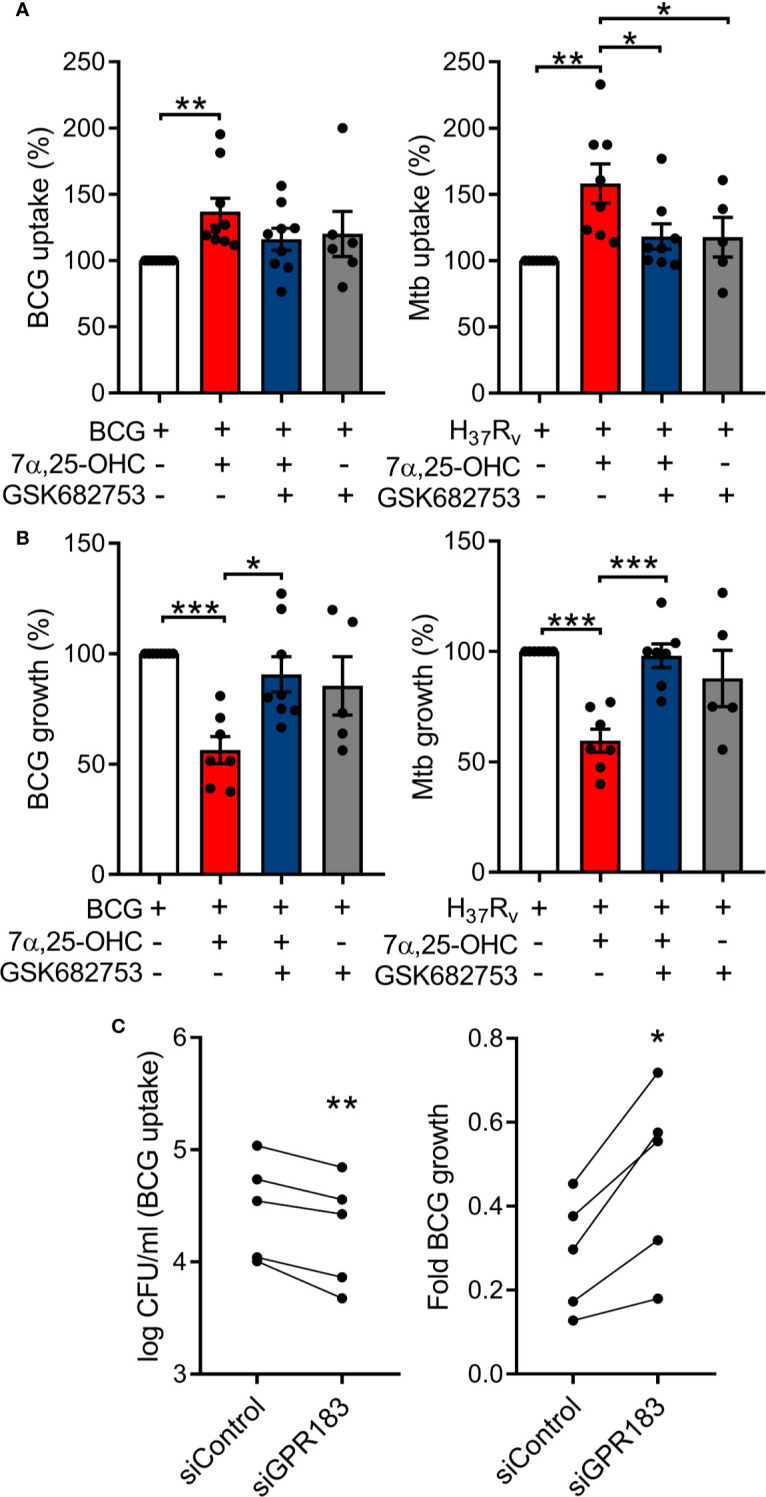
Oxysterol-induced activation of GPR183 in primary MNs significantly inhibits intracellular mycobacterial growth, while GPR183 knockdown increases intracellular mycobacterial growth. Primary MNs from eight donors **(A)** and seven donors **(B)** were infected with BCG or Mtb H37Rv (MOI 1), ± 7α,25-OHC (100 nM), ± GSK682753 (10 µM). Uptake of **(A)** BCG and Mtb H37Rv was determined at 2h p.i. Growth of **(B)** BCG and Mtb H37Rv was determined at 48h post-infection. Percent of mycobacterial growth was calculated as the fold change of CFU at 48h compared to CFU at 2h, normalized to non-treated cells. PMA-differentiated THP-1 cells were transfected with 20 nM of either negative control siRNA or GPR183 siRNA for 48h before infection with BCG (MOI 1). **(C)** Mycobacterial uptake was determined at 2h and intracellular mycobacterial growth was determined at 48h p.i. (normalized to uptake). Data are presented as means ± SEM; *P ≤ 0.05; **P ≤ 0.01; ***P ≤ 0.001; paired t-test.

We investigated whether *in vitro* activation of GPR183 with its endogenous agonist impacts the immune response to mycobacteria in primary human MNs. MNs from 15 healthy donors were infected with BCG (n = 7) or Mtb H_37_R_V_ (n = 8) (**Figure 2**) at a MOI of one in the presence or absence of the GPR183 agonist 7*α*,25-OHC and/or the antagonist GSK682753. Activation of GPR183 by 7*α*,25-OHC significantly increased the uptake of BCG and Mtb H_37_R_V_ (**Figure 2A**) at 2 h p.i. This increase in phagocytosis was abolished by the simultaneous addition of the GPR183 antagonist GSK682753, confirming that increased mycobacterial uptake was the result of GPR183 activation. Interestingly, we observed ~50% reduction in the growth of BCG and Mtb H_37_R_V_ (**Figure 2B**) by 48h p.i. in 7*α*,25-OHC treated cells, and again, this effect was abrogated by GSK682753. The addition of 7*α*,25-OHC and/or GSK682753 had no detrimental effect on the viability of human THP-1 cells (**Supplementary Figure 2A**). There was also no effect of 7*α*,25-OHC and GSK682753 on BCG growth in liquid culture (**Supplementary Figure 2B**), thus confirming that the significant mycobacterial growth inhibition in MN cultures was attributable to the immune modulatory activity of 7*α*,25-OHC *via* GPR183. Independently, we observed that H_37_R_v_ down-regulates GPR183 in primary MNs (**Supplementary Figure 3**).

To confirm the role of GPR183 in phagocytosis and growth inhibition, we next performed GPR183 siRNA knockdown experiments. Differentiated THP-1 cells were transfected with 20 nM of *GPR183*-targeting siRNA (siGPR183) or negative control siRNA (siControl). We observed ~80% reduction of *GPR183* mRNA level and ~50% reduction of protein expression in cells transfected with siGPR183 when compared to siControl-transfected cells (**Supplementary Figures 4A, B**) at 48 h. Forty-eight hours after transfection the cells were infected with BCG at a MOI of one. We observed a marked decrease in BCG uptake in cells transfected with siGPR183 (p = 0.0048) compared to siControl-transfected cells and a significant increase in intracellular mycobacterial growth over time (p = 0.0113, **Figure 2C**).

### GPR183 Is a Negative Regulator of the Type I Interferon Pathway in Human MNs

**Figure 3:**
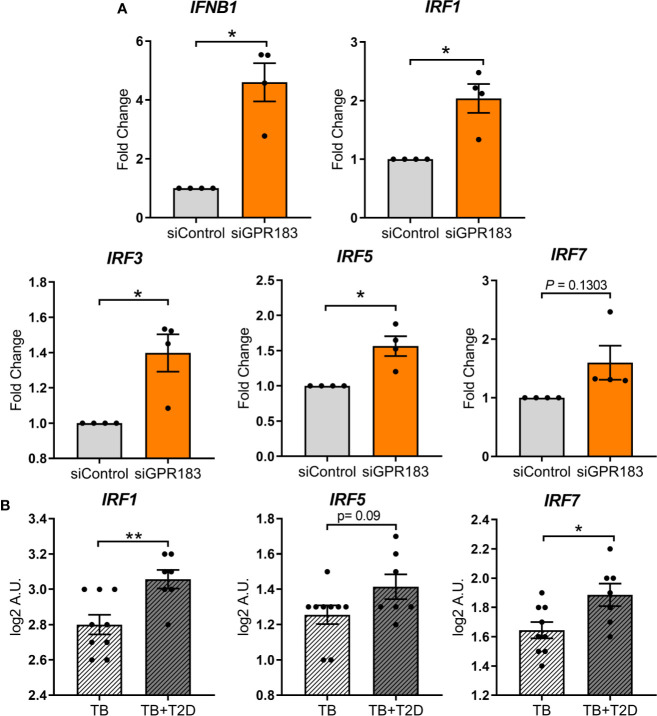
GPR183 knockdown increases expression of transcription factors regulating type I interferon responses. **(A)** Total RNA was isolated from primary MNs following 48 h incubation with 20 nM GPR183 siRNA (or negative control siRNA). Gene expression of *IFNB1*, *IRF1*, *IRF3*, *IRF5, IRF7* was measured by qRT-PCR using RPS13 as reference gene. Data are normalized to cells transfected with negative control siRNA. **(B)** NanoString analyses of RNA isolated from TB and TB + T2D cohort showed similar increase in type I IFN associated genes *IRF1*, *IRF5*, *IRF7*. Data are presented as fold changes ± SEM; *P ≤ 0.05; **P ≤ 0.01; paired *t*-test.

In genome wide association studies GPR183 has been implicated as a negative regulator of the IRF7 driven inflammatory network (25). Therefore, we focused subsequent experiments on type-I IFN regulation. To determine whether GPR183, a constitutively active GPCR (26), has a direct effect on *IRFs* and *IFNB1* expression, we performed knockdown experiments in primary MNs. GPR183 knockdown (**Supplementary Figure 4C**) up-regulated *IFNB1* (2.7–5.5 fold; *P* = 0.0115) as well as *IRF1, IRF3, IRF5*, and *IRF7*, although the latter did not reach statistical significance (**Figure 3A**).

*IRF1*, *IRF5*, and *IRF7* transcripts were similarly up-regulated in whole blood from TB + T2D patients compared to TB patients (**Figure 3B**), consistent with the downregulation of *GPR183* mRNA expression (**Figure 1**). *IRF3* expression was not significantly different between TB and TB + T2D patients (data not shown).

### GPR183 Activation Induces a Cytokine Profile Favoring Mtb Control

**Figure 4:**
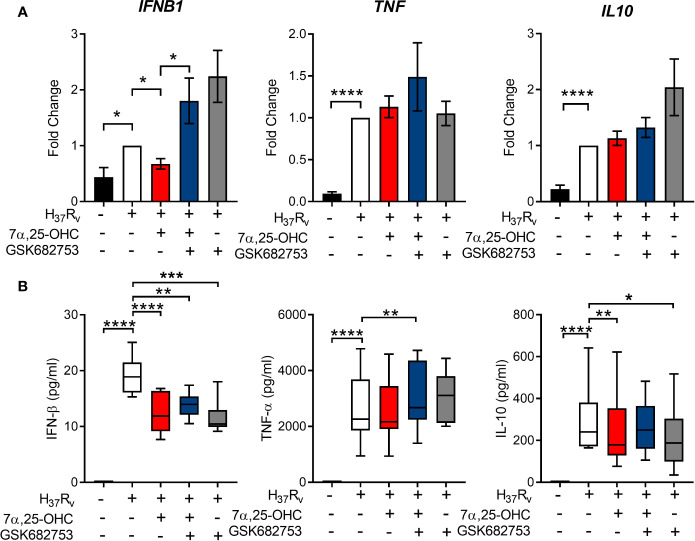
Activation of GPR183 leads to cytokine production favoring Mtb control. Primary MN from healthy donors (n = 8) were infected for 2 h with Mtb H37Rv (MOI 10:1), 7a,25-OHC (100 nM), and/or GSK682753 (10 μM). Cells were washed and left with drugs for a further 22 h. Changes in the expression of **(A)** IFNB1, TNF, and IL10 were measured by qPCR and normalized to untreated infected cells. Concentrations of **(B)** IFN-b, TNF-a, and IL-10 in the culture supernatant were measured by ELISA. Data are presented as mean fold change ± SEM or min to max for box plots; *P ≤ 0.05; **P ≤ 0.01; ***P ≤ 0.001; ****P ≤ 0.0001; paired t-test.

Next, we investigated whether the reduced intracellular mycobacterial growth observed in primary MNs treated with 7*α*,25-OHC was associated with a change in MN secreted cytokines. Gene expression of *IFNB1, TNF*, and *IL-10* was measured 24 h following infection with Mtb H_37_R_V_ at MOI of one (**Figure 4A**). The concentrations of the corresponding cytokines were measured in cell culture supernatant by ELISA (**Figure 4B**). Mtb infection significantly up-regulated the expression of *IFNB1* (*P* = 0.0068), *TNF* (*P* = 0.0001), *IL-10* (*P* < 0.0001) (**Figure 4A**), and *IL-1B* (**Supplementary Figure 5A**). 7*α*,25-OHC significantly down-regulated Mtb-induced *IFNB1* expression (*P* = 0.0017), while it did not affect *TNF*, *IL-10* or *IL-1B* expression. At the protein level, the concentrations of IFN-β and IL-10, but not TNF-α (*P* < 0.0001 and *P* = 0.0090, respectively, **Figure 4B**) nor IL-1β (**Supplementary Figure 5B**) were significantly lower in the culture supernatant of 7*α*,25-OHC-treated Mtb-infected primary MNs compared to untreated infected cells.

### The Oxysterol 7*α*,25-OHC Induces Autophagy

**Figure 5:**
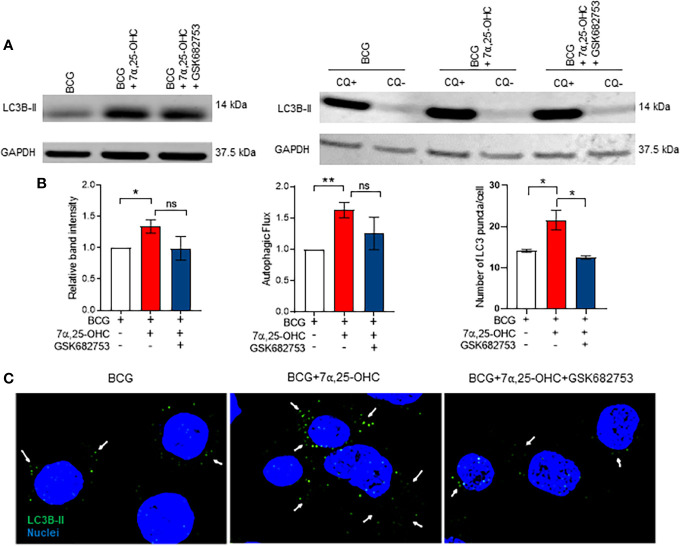
Treatment with 7*α*,25-OHC induces autophagy. PMA-differentiated THP-1 cells were infected/uninfected and co-incubated with ±7*α*,25-OHC, ±GSK682753, for 2 h. Extracellular BCG was removed, and cells were incubated for a further 4 h or 22 h in RPMI medium containing drugs. **(A)** Cells were lysed at 6 h or 24 h (Flux) p.i. **(B)** The band intensity was then normalized to the reference protein, GAPDH and further normalized to the BCG. Autophagic flux was obtained by subtracting chloroquine positive values with chloroquine negative values. **(C)** Cells were visualized using the Olympus FV 3000 confocal microscope. At least 30 cells were counted for every condition. Data are presented as ± SEM; ns, *P* > 0.05; *P ≤ 0.05; **P ≤ 0.01; unpaired *t*-test.

We aimed to identify whether 7*α*,25-OHC impacts the production of reactive oxygen species (ROS) and the autophagy pathway. ROS production in BCG-infected primary MNs was not affected by 7*α*,25-OHC (**Supplementary Figure 6**); however, we observed an increase in accumulation of the autophagosome marker LC3B-II in BCG-infected THP-1 cells treated with 7*α*,25-OHC (*P* = 0.0119, **Figure 5A**). We next performed the experiments in the absence and presence of the lysosomal inhibitor chloroquine in order to determine autophagic flux. Autophagic flux in BCG-infected cells was significantly increased with 7*α*,25-OHC treatment (*P* = 0.0069, **Figure 5B**). The simultaneous addition of the GPR183 antagonist GSK682753 with 7*α*,25-HC, decreased the levels of LC3B-II and autophagic flux; however, this did not reach statistical significance.

We next confirmed the induction of autophagy *via* microscopy. The number of LC3B-II puncta per cell increased in 7*α*,25-OHC stimulated BCG-infected THP-1 cells compared to the untreated BCG-infected cells (*P* = 0.0358, **Figure 5C**). The 7*α*,25-OHC effect could be reduced by antagonist GSK682753 (*P* = 0.0196).

### GPR183 KO Mice Have Higher Bacterial Burden During the Early Stage of Infection

**Figure 6:**
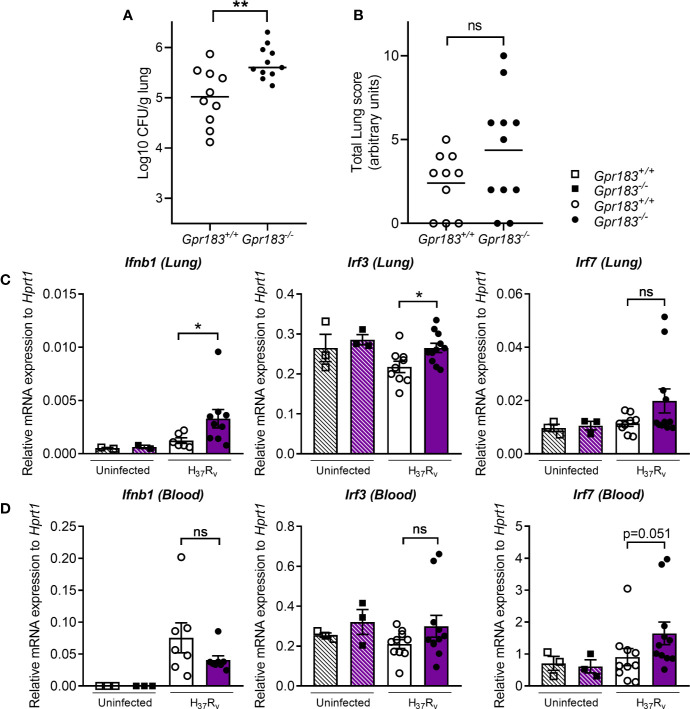
GPR183KO mice have higher lung CFU, corresponding with increased expression of transcription factors regulating type I interferon responses. Mice were infected with 300 CFU of aerosol Mtb H_37_R_v_. **(A)** Bacterial lung burden 2 weeks p.i. **(B)** Total histology lung score. RNA was isolated from Mtb-infected lung and blood samples 2 weeks p.i. **(C)** Gene expression of *Ifnb1*, *Irf3*, and *Irf7* in the lungs, **(D)** *Ifnb1, Irf3*, and *Irf7* in the blood, was measured by qRT-PCR using *Hprt1* as reference gene. Data are presented as ± SEM; ns, *P* > 0.05; *P ≤ 0.05; **P ≤ 0.01.

**Figure 7:**
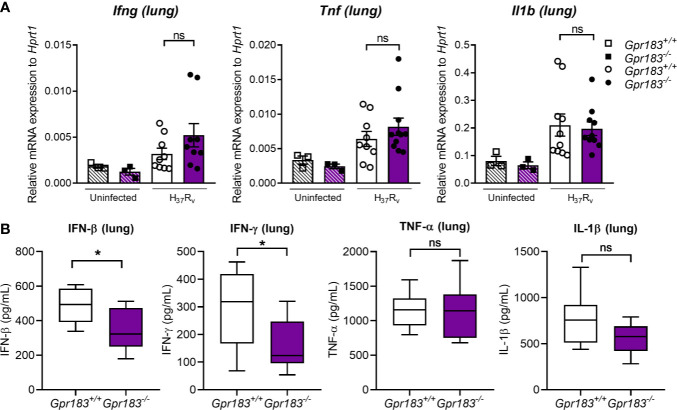
Pro-inflammatory cytokine expression at 2 weeks p.i. of Mtb H_37_R_v_-infected mice. Mice were infected with 300 CFU of aerosol Mtb H_37_R_v_. **(A)** Gene expression of *Ifng*, *Il1b*, and *Tnf* in the lungs. **(B)** Concentrations of IFN-β, IFN-**γ**, IL-1β and TNF-α in the culture supernatant were measured by ELISA. Data are presented as ± SEM; ns, *P* > 0.05; *P ≤ 0.01.

To confirm the effect of the GPR183 receptor *in vivo*, we infected WT and GPR183 KO mice with aerosolized Mtb. At 2 weeks p.i., GPR183 KO mice showed significantly increased mycobacterial burden in the lungs compared to WT mice (*P* = 0.0084, **Figure 6A**), while the bacterial burden was comparable at 5 weeks p.i. (**Supplementary Figure 7**). GPR183 KO mice also had higher lung pathology scores although this did not reach significance (**Figure 6B**). GPR183 KO mice had significantly increased *Ifnb1* expression in the lungs (*P* = 0.0256; **Figure 6C**), along with increased *Irf3* (*P* = 0.0159); however, *Irf5* (**Supplementary Figure 8**) and *Irf7* (**Figure 6C**) remained unchanged. *Irf7* transcription was increased in the blood from GPR183 KO compared to WT mice (*P* = 0.0513; **Figure 6D**), but *Ifnb1*, *Irf3* and *Irf5* expression was not different (**Figure 6D**, **Supplementary Figure 6**). At the RNA level *Tnf*, *Ifng*, and *Il1b* were not significantly different between GPR183 KO and WT mice (**Figure 7A**). Unexpectedly, at the protein level, the concentrations of IFN-β (*P* = 0.0232) and IFN-*γ* (*P* = 0.0232) were significantly lower in GPR183 KO mice lung, while TNF-α (*P* = 0.7394) and IL-1β (*P* = 0.0753) were not statistically different (**Figure 7B**).

## Discussion

Historically oxidized cholesterols, so called oxysterols, were considered by-products that increase polarity of cholesterol to facilitate its elimination. However, they have recently emerged as important lipid mediators that control a range of physiological processes including metabolism, immunity, and steroid hormone synthesis (27).

**Figure 8:**
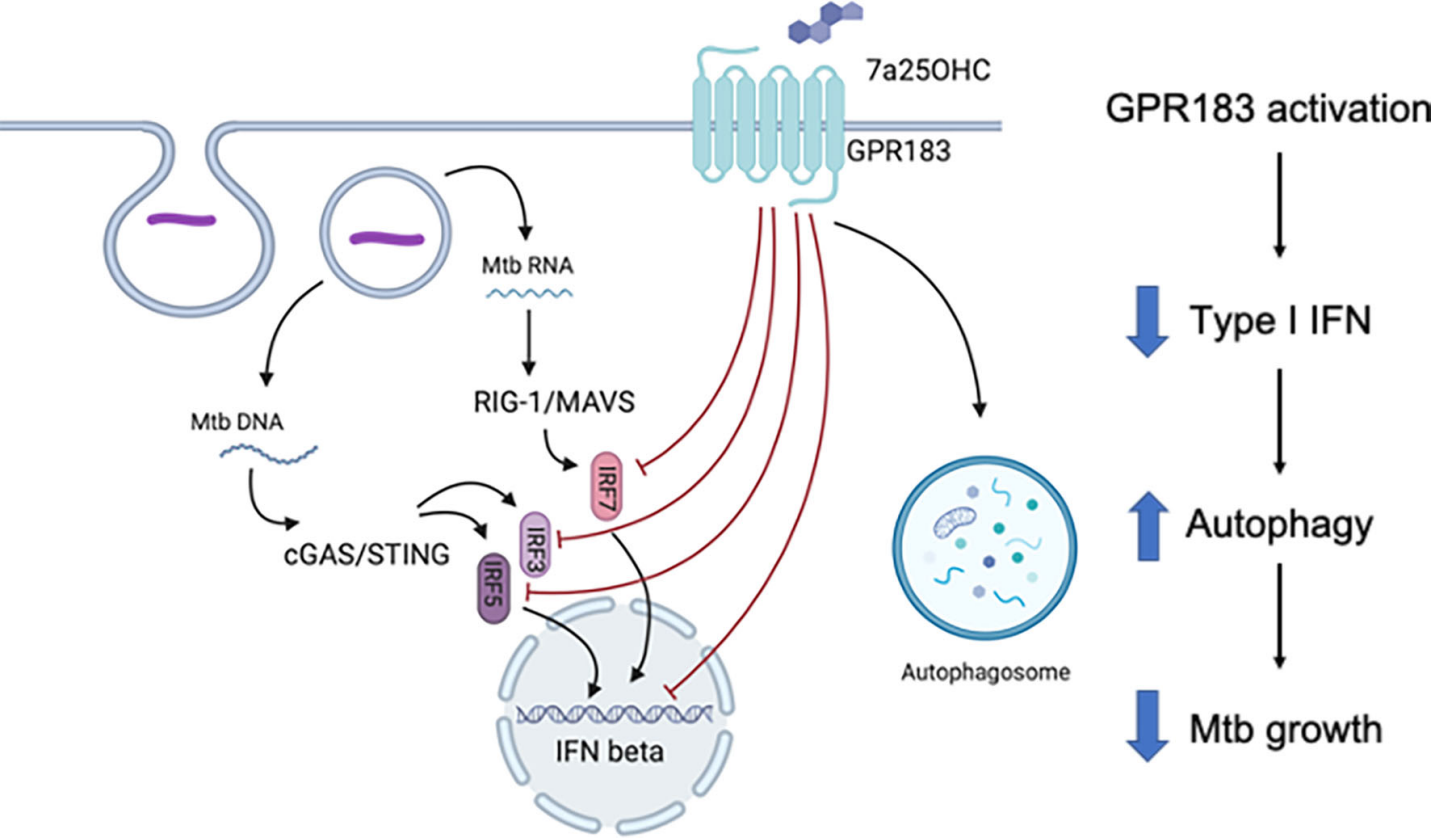
Schematic summary of the role of GPR183 in Mtb-infected human monocytes.

Our findings define a novel role for GPR183 in regulating the host immune response during Mtb infection (summarized in **Figure 8**). We initially identified GPR183 through a blood transcriptomic screen in TB and TB + T2D patients and found an inverse correlation between GPR183 expression and TB disease severity on chest x-ray. Although we demonstrate that the decrease in blood GPR183 in TB + T2D patients is likely due, in part, to a decreased frequency of non-classical monocytes expressing GPR183, we cannot rule out that reduced GPR183 expression in whole blood is partially attributable to neutrophils and eosinophils as preferential loss of neutrophils and eosinophils occurs upon PBMC isolation. In our study the TB patients with T2D had more severe TB compared to those without T2D; therefore we cannot ascertain whether lower GPR183 expression is linked to TB + T2D comorbidity or TB disease severity.

We demonstrate that activation of GPR183 by 7*α*,25-OHC in primary human MNs during Mtb infection results in significantly better control of intracellular Mtb growth. This is in contrast to a recently published study showing increased Mtb growth with 7*α*,25-OHC when added post-infection in murine RAW264.7 cells (28). The discrepancies between the studies could also be attributed to the different cell types and infection dose, which was 25 times higher in the aforementioned study. Consistent with the findings of Tang et al. (28) in murine cells we show that mycobacterial infection down-regulates GPR183 in human MNs, which may be an immune-evasion strategy specific to mycobacteria since LPS, a constituent of Gram-negative bacteria, upregulates GPR183 (15). Whether the observed increase in phagocytosis in the presence of 7*α*,25-OHC is a non-specific effect driven by internalization of agonist bound GPR183 and non-specific uptake of bacteria or an increase in pattern recognition receptors remains to be elucidated.

We further demonstrate that GPR183 activation by 7*α*,25-OHC reduces IFN-β expression and secretion in Mtb-infected primary MNs and targeted GPR183 knockdown significantly upregulating *IRFs* and *IFNB1*. Similarly, gene expression of *IRF1*, *IRF5*, and *IRF7* is up-regulated in TB + T2D patients compared to TB patients and corresponds with down-regulation of *GPR183*, thereby demonstrating that GPR183 expression is associated with IFN regulatory factors during human TB, and GPR183 is a negative regulator of type I IFNs in Mtb-infected human MNs.

There is mounting evidence that the production of type-I IFNs is detrimental during Mtb infection (29, 30). Up-regulation of type-I IFN blood transcript signatures occurs in TB disease and correlates with disease severity (31). In macrophages, Mtb induces up-regulation of *IFNB1* expression as early as 4 h p.i. to limit IL-1β production, a critical mediator in the host defense against Mtb (32). Although 7*α*,25-OHC significantly reduced *IFNB1* mRNA, we did not observe an increase in *IL1B* mRNA, suggesting that the GPR183-mediated regulation of type-I IFN does not influence IL1B expression. In addition to GPR183 mediated reduction in IFN-β, we observed a decrease in IL-10 in Mtb-infected primary MNs treated with 7*α*,25-OHC. IL-10 production is induced by type-I IFN signaling (33, 34) and promotes Mtb growth (35) by reducing the bioavailability of TNF-α through the release of soluble TNF receptors and preventing the maturation of Mtb-containing phagosomes (35–38). Collectively, we show that GPR183 is a negative regulator of type-I IFNs in primary MNs, and agonist induced activation of GPR183 reduces Mtb-induced IFN-β production, while leaving expression of cytokines important for Mtb control unchanged.

Further confirming the role of GPR183, GPR183 KO mice infected with Mtb had significantly higher bacterial burden in the lung compared to WT mice 2 weeks p.i. (prior to initiation of the adaptive immune response to Mtb) with this effect disappearing at 5 weeks p.i., when T cell responses against Mtb are fully established. Our results thus strengthen the contention that GPR183 plays an important role in the innate immune control of Mtb irrespective of hyperglycemia. We confirmed the importance GPR183 in regulating type-I interferons during Mtb infection *in vivo*. GPR183 KO mice infected with Mtb had significantly increased lung *Ifnb1* and *Irf3* mRNA. Unexpectedly, IFN-β and IFN-**γ** secretions were both significantly downregulated in the lung. These differences between mRNA and protein levels may be due to kinetic parameters of transcription *versus* translation or mRNA stability *versus* protein consumption.

Furthermore, we demonstrate that the GPR183 agonist 7*α*,25-OHC promotes autophagy in macrophages infected with mycobacteria. Autophagy is a cellular process facilitating the elimination of intracellular pathogens including Mtb (39). Antimicrobial autophagy was shown to be inhibited by *Mycobacterium leprae* through upregulation of IFN-β and autocrine IFNAR activation which in turn increased expression of the autophagy blocker OASL (2′-5′-oligoadenylate synthetase like) (40). Whether there is a link between the 7*α*,25-OHC-induced reduction of IFN-β production and the increase in autophagy remains to be investigated in future studies.

Several autophagy promoting re-purposed drugs including metformin are currently being assessed as HDTs for TB (41). We propose that GPR183 is a potential target for TB HDT, warranting the development of specific, metabolically stable small-molecule agonists for this receptor to ultimately improve TB treatment outcomes in TB patients with and without T2D co-morbidity.

## Data Availability Statement

The raw data supporting the conclusions of this article will be made available by the authors, without undue reservation.

## Ethics Statement

The human studies were approved by the Institutional Review Board of Stellenbosch University (N13/05/064 and N13/05/064A), and all study participants signed pre-approved informed consent documents prior to enrolment into the studies. The patients/participants provided their written informed consent to participate in this study. All animal studies were approved by the Animal Ethics Committee of the University of Queensland (MRI-UQ/596/18) and conducted in accordance with the Australian Code for the Care and Use of Animals for Scientific Purposes.

## Author Contributions

AG, SB, and KR wrote the manuscript. AG, SB, RS, SH, HS, MN, CF, LK, HT, TW, HB-O, AH, CM, LV, and NW carried out the experiments. AG, SB, MN, HS, RS, and SH analyzed the data. TM-P, MR, LS, GW, and KR interpreted the data and developed the theoretical framework. KR conceived the original idea. All authors contributed to the article and approved the submitted version.

## Funding

This study was supported by the National Institutes of Health (NIH), National Institute of Allergy and Infectious Diseases (NIAID) and the South African Medical Research Council under the US-South African Program for Collaborative Biomedical Research (grant number: R01AI116039) to KR and by the TANDEM Grant of the EUFP7 (European Union’s Seventh Framework Program) under Grant Agreement NO. 305279 to GW for study participant recruitment, by the Novo Nordisk Foundation to MR and TM-P. All other laboratory-based research activities were supported by grants from the Australian Infectious Diseases Research Center, The Australian Respiratory Council and the Mater Foundation to KR. The Translational Research Institute is supported by a grant from the Australian Government.

## Conflict of Interest

MMR is a co-founder of Antag Therapeutics and of Synklino.

The remaining authors declare that the research was conducted in the absence of any commercial or financial relationships that could be construed as a potential conflict of interest.
